# Anesthetic management of a child with congenital insensitivity to pain with anhidrosis: A case report

**DOI:** 10.3389/fsurg.2022.997162

**Published:** 2022-09-12

**Authors:** Ying Zhang, Zhiyu Geng

**Affiliations:** Department of Anesthesiology, Peking University First Hospital, Beijing, China

**Keywords:** congenital insensitivity to pain with anhidrosis, autonomic neuropathy type VI, anesthesia, pediatrics, laryngeal mask airway

## Abstract

Congenital insensitivity to pain with anhidrosis (CIPA) is a rare, autosomal recessive disease classified as hereditary sensory and autonomic neuropathy type VI. Patients with CIPA are characterized by insensitivity to pain, episodes of unexplained fever, anhidrosis, self-mutilating behavior, intellectual disability, and autonomic nervous system abnormalities. The clinical features may intrinsically pose anesthetic challenges. We present a case of a patient with CIPA who underwent tumor biopsy under general anesthesia using a Supreme laryngeal mask airway without any complications. The anesthetic management of this condition is discussed.

## Introduction

Congenital insensitivity to pain with anhidrosis (CIPA), also known as hereditary sensory and autonomic neuropathy type IV (HSAN IV), is a rare, autosomal recessive disorder (1). The clinical presentation of CIPA is characterized by a lack of response to painful stimuli, anhidrosis, recurrent episodic fever, intellectual disability, and autonomic nervous system abnormalities (1–3). Patients with CIPA are prone to self-harm because of pain insensitivity and intellectual disability. They may require multiple surgeries throughout their lifetime due to fractures, trauma, infections, osteomyelitis, and joint deformities. Anesthesia management for patients with CIPA is challenging. Some studies have reported perioperative regurgitation, aspiration, hyperthermia, and cardiovascular complications in these patients (4, 5). We herein report a case of a child patient with CIPA who successfully underwent general anesthesia with a Supreme laryngeal mask airway (LMA). We obtained written informed consent from the patient’s parents for the procedure and publication.

## Case report

An 8-year-old boy, height 135 cm, weight 24.5 kg, was admitted to our hospital with a fever lasting more than 12 days. His body temperature ranged from 38.5 to 40 °C, with fever peaks twice daily. He was discovered to be insensitive to pain and unable to sweat after birth. Genetic testing revealed a mutation of the neurotrophic tyrosine receptor kinase 1 (*NTRK1*) gene, and CIPA was diagnosed when he was 7 years old. Other characteristics of the patient were intellectual disability, dry skin, unsteady walking, swelling of the left hip, and short left lower extremity. His preoperative hemoglobin was 8.8 g/dl. Ultrasound and magnetic resonance imaging demonstrated dislocation of the left hip joint and a cystic and solid mass around the left hip joint, approximately 10.5 cm × 5.9 cm in size, with abundant blood flow inside, and Ewing's sarcoma with aneurysmal bone cyst was considered. A diagnostic tumor biopsy under general anesthesia was scheduled.

In the operating room, standard monitoring was applied, including blood pressure, electrocardiogram, pulse oximeter, and bispectral index (BIS). His tympanic temperature was 37.0 °C. The restless patient was sedated with intravenous midazolam 1 mg and dexmedetomidine 1 µg/kg infused within 10 min. Anesthesia was induced with intravenous propofol 50 mg, sufentanil 2 µg, and sevoflurane 2% inhaled *via* a facemask. A 2.5 Supreme LMA was inserted, and the patient was breathing spontaneously. The patient was placed in a lateral decubitus position during the surgery. Maintenance of anesthesia was achieved with sevoflurane 1%–2% in a 50% oxygen/air mixture. End-tidal carbon dioxide and BIS were continuously monitored. No muscle relaxant was given, and vital signs were stable throughout the operation. The operation room temperature was kept at around 22 °C. His tympanic temperature was monitored with an infrared thermometer. The patient’s body temperature was stable, and we took no warming or cooling measures intraoperatively. The surgery lasted for 40 min. At the end of the operation, sevoflurane was stopped, and the LMA was removed. Recovery was uneventful, and the patient’s tympanic temperature was 36.6 °C. He was transferred to the ward in stable condition. The pathohistology result showed a benign lesion, and he was discharged home with an almost normal temperature 7 days later.

## Discussion

Congenital insensitivity to pain with anhidrosis is an autosomal recessive disease caused by mutations in the *NTRK1* gene, which encodes tropomyosin-related kinase receptor A, a high-affinity receptor for the nerve growth factor (NGF) (1). Mutations in the *NTRK1* gene cause apoptosis of NGF-dependent nociceptive sensory neurons and autonomic sympathetic neurons during embryonic development, leading to a lack of nociceptive reception (6). Patients were characterized by recurrent episodic fevers, anhidrosis, insensitivity to pain, and self-mutilating behavior. Autonomic nervous system dysfunction makes both general and neuraxial anesthesia challenging.

For patients who can cooperate, neuraxial anesthesia can be used for abdominal and lower extremity surgeries. Pirani et al. (7) reported that a parturient with CIPA successfully underwent neuraxial anesthesia for cesarean delivery. Our patient had intellectual disability, was irritable, and was unable to cooperate, so we ultimately chose general anesthesia for this diagnostic procedure. Before induction, intravenous midazolam and dexmedetomidine were administered to calm this restless patient.

Despite a lack of peripheral pain sensation, CIPA patients still respond to airway manipulation, and a certain depth of anesthesia is needed to reduce the hemodynamic response to endotracheal intubation (8). Therefore, in this diagnostic procedure, we used a laryngeal mask airway instead of tracheal intubation to mitigate hemodynamic stimulation.

Due to autonomic nervous system dysfunction, CIPA patients may be predisposed to gastroparesis, delayed gastric emptying, and an increased risk for regurgitation and aspiration. Zlotnik et al. (9) reported two cases of intraoperative regurgitation and aspiration with LMA and recommended that patients with CIPA should be considered as having a “full stomach” and rapid sequence induction with an endotracheal tube should be utilized in all CIPA patients. However, in this case, we used the Supreme laryngeal mask for airway management. As one of the second-generation LMAs, it has a gastric drain tube and higher oropharyngeal leak pressure, which could provide a satisfactory airway for positive pressure ventilation. The correct Supreme laryngeal mask position is vital to achieving a good seal to prevent fluid in the hypopharynx from entering the airway. We did not detect any clinical evidence of regurgitation or aspiration, and the postoperative recovery was unremarkable.

A primary anesthesia concern with CIPA is impaired body temperature control. Although hyperthermia is seldom reported in the literature, body temperature should be carefully monitored and controlled with cooling or warming blankets if necessary (5). The operating room temperature should be adjusted appropriately. In addition, the genetic anomalies responsible for malignant hyperthermia and CIPA are different, and succinylcholine and halogenated agents can be used for CIPA patients. In our case, body temperature was monitored, the initial temperature was 37.0 °C, and the temperature decreased to 36.6 °C by the end of surgery.

Dexmedetomidine is a highly selective α_2_-agonist with sedative and analgesic effects and minimal respiratory depression. These favorable characteristics make it potentially useful for anesthesia in pediatric surgeries (10, 11). In our case, 1 µg/kg dexmedetomidine administered intravenously before induction made the hemodynamic status more favorable when combined with sevoflurane 1%–2% intraoperatively. Hypotension and bradycardia were not observed during the operation, and the patient recovered without emergence agitation.

## Conclusion

This case demonstrated that for patients with congenital insensitivity to pain with anhidrosis, the Supreme LMA and dexmedetomidine-sevoflurane-based anesthesia might be safely used for minor surgery. Body temperature and end-tidal carbon dioxide should be routinely monitored.

## Data Availability

The original contributions presented in the study are included in the article/Supplementary Material, further inquiries can be directed to the corresponding author/s.
